# Improving the Activity and Selectivity of a Scorpion-Derived Peptide, A3a, against *Acinetobacter baumannii* through Rational Design

**DOI:** 10.1021/acsomega.4c09593

**Published:** 2025-01-30

**Authors:** Dalton S. Möller, Mandelie van der Walt, Carel Oosthuizen, Miruna Serian, June C. Serem, Christian D. Lorenz, A. James Mason, Megan J. Bester, Anabella R. M. Gaspar

**Affiliations:** † Department of Biochemistry, Genetics and Microbiology, Faculty of Natural and Agricultural Sciences, University of Pretoria, Pretoria 0002, South Africa; ‡ Drug Discovery and Development Centre (H3D), University of Cape Town, Rondebosch 7701, South Africa; § Department of Physics, Faculty of Natural, Mathematical and Engineering Sciences, King’s College London, London WC2R 2LS, U.K.; ∥ Department of Anatomy, Faculty of Health Sciences, University of Pretoria, Pretoria 0002, South Africa; ⊥ Department of Engineering, Faculty of Natural, Mathematical and Engineering Sciences, King’s College London, London WC2R 2LS, U.K.; # Institute of Pharmaceutical Science, School of Cancer & Pharmaceutical Science, Faculty of Life Sciences & Medicine, King’s College London, London SE1 9NH, U.K.

## Abstract

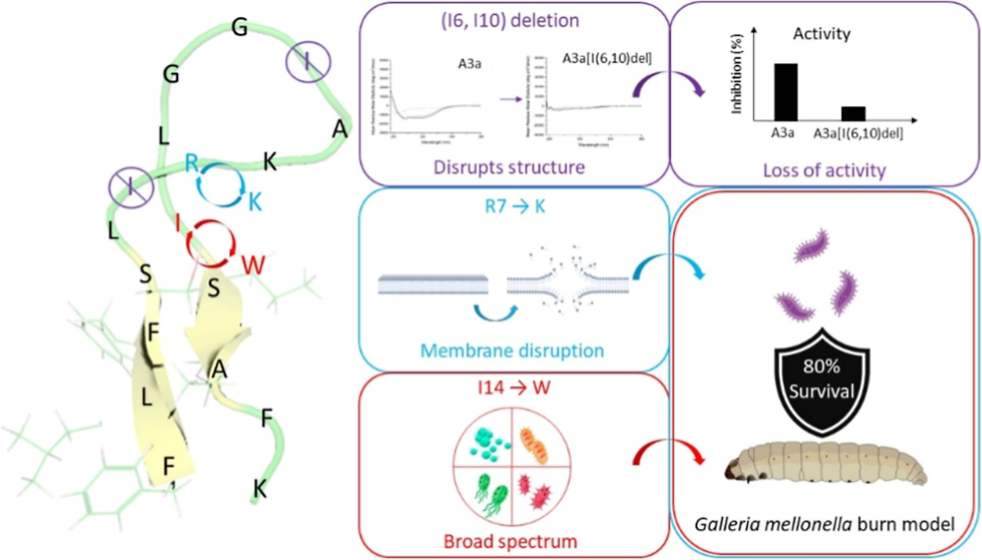

The rise in antimicrobial resistance has led to an increased desire to understand how antimicrobial peptides (AMPs) can be better engineered to kill antibiotic-resistant bacteria. Previously, we showed that C-terminal amidation of a peptide, identified in scorpion *Androctonus amoreuxi* venom, increased its activity against both Gram-positive and -negative bacteria. Here, we incorporate all-atom molecular dynamics (MD) simulations in a rational design strategy to create analogues of A3a with greater therapeutic potential. We discover two novel AMPs which achieve greater potency against, and selectivity toward, *Acinetobacter baumannii* ATCC 19606 but via two distinct mechanisms and which are effective in *Galleria mellonella* models of *A. baumannii* burn wound infection. While CD spectroscopy indicates A3a adopts an α-helix conformation in the presence of models of the Gram-negative bacterial plasma membrane, MD simulations reveal it adopts a hairpin conformation during initial binding. Three different strategies, designed to stabilize this hairpin conformation, produce substantially different outcomes. Deletion of Ile6 and Ile10 restricts conformational flexibility, characteristic of A3a, during membrane binding, prevents adoption of the α-helix conformation in the steady state, and abrogates the antibacterial activity. In contrast, substitution of arginine 7 to lysine (A3a[R7K]) or isoleucine 14 to tryptophan (A3a[I14W]) does not consistently affect peptide conformations. Both of these new analogues are rapidly bactericidal toward *A. baumannii* ATCC 19606 but A3a[R7K] also causes rapid permeabilization and while the antibacterial potency and selectivity are increased for both peptides, this is greatest for A3a[I14W]. Integration of atomistic MD simulations into a multidisciplinary approach to understanding antimicrobial peptide mechanism of action is a valuable tool for interpreting the effects of rational design strategies.

## Introduction

The worldwide misuse of antibiotics has led to an increase in antibiotic resistance, and consequently, the design and discovery of new antibiotics is vital. The associated pathogens, referred to as the ESKAPE pathogens, are a list of highly virulent and antibiotic resistant bacterial pathogens that includes *Enterococcus faecium*, *Staphylococcus aureus*, *Klebsiella pneumoniae*, *Acinetobacter baumannii*, *Pseudomonas aeruginosa*, and *Enterobacter* spp.^1^ The acronym ESKAPEE is sometimes used to include *Escherichia coli.* Of these six ESKAPE pathogens, four are Gram-negative bacteria, emphasizing the need for antibiotics that specifically target Gram-negative pathogens.

The development of antimicrobials against Gram-negative bacteria is challenging due to the greater complexity of the cell wall when compared with Gram-positive bacteria. In Gram-negative bacteria, the cell wall consists of an outer membrane that provides additional protection and the presence of lipopolysaccharides (LPSs) increases the stability and charge of the cell wall.^2^ In addition, enzymes within the outer lipid bilayer that includes proteases (OmpT),^3^ a LPS-modifying enzyme (PagP),^4^ and phospholipase (PldA),^5^ provide further protection. Consequently, the opportunity for antibiotic resistance to develop is increased, as it is more difficult for conventional antibiotics to reach their intracellular targets. Therefore, it is important to develop antibiotics that effectively permeate or disrupt both inner and outer membranes to effect rapid cell death.

Antimicrobial peptides (AMPs) are key components of the innate immune system of living organisms. Due to their multifunctionality and diversity in both sequence and modes of action,^6^ AMPs have become leading compounds for the development of new peptide-based antibiotics. Compared with conventional antibiotics, naturally occurring peptides come with limitations including susceptibility to protease activity, higher costs of production, and lower selectivity. To overcome these limitations, researchers are increasingly using rational drug design^7−9^ with the aim of improving AMPs by manipulating certain physiochemical properties, such as adding residues with positively charged side chains to improve selectivity^10^ or hydrophobic residues which increase membrane insertion and subsequent activity.^11^

The AMP, A3 (FLFSLIRKAIGGLISAFK), was originally derived from AamAP1, a host-defense peptide identified in the venom of a North African scorpion *Androctonus amoreuxi*. Previous studies show AamAP1 has moderate activity, with minimum inhibitory concentrations (MICs) of 150 μM and 20 μM against *E. coli* and *S. aureus*, respectively, but causes the hemolysis of horse erythrocytes.^12^ To improve activity and reduce hemolytic activity, A3 was generated from AamAP1 by substituting proline 7 with arginine and histidine 8 with lysine.^13^ The activity of this analogue increased against Gram-positive bacteria, with MIC values ranging between 5 and 15 μM against *S. aureus*, *Enterococcus faecalis*, *Staphylococcus epidermidis*, and *E. faecium*. The amount of hemolysis also decreased, with 40 μM causing 36.1% hemolysis. However, the activity of A3 against Gram-negative bacteria was still limited.

Previously, we amidated the C-terminus of A3 to improve its activity against Gram-negative bacteria.^14^ A3, the amidated analogue A3a, and the parent peptide AamAP1 were evaluated for antibacterial activity against a panel of ESKAPE pathogens. A3a was active against Gram-positive bacteria (MIC ranging from 4 to 16 μM) with increased activity against Gram-negative bacteria (MIC ranging from 4 to 8 μM). In the previous QSAR study where we considered a range of parameters associated with cytotoxicity, including increased overall charge, the minimum concentration needed to cause 50% inhibition (IC_50_) of HaCat cell viability was 59.9 μM and 147.2 μM for A3 and A3a, respectively, indicating >2-fold decease in the cytotoxicity of A3a compared with A3.^14^ The increased selectivity of A3a for Gram-negative bacteria identified it as a promising candidate for use as a template in rational design.

The activity and cytotoxicity of AMPs are strongly associated with their secondary structures. Due to the nature of how α-helical peptides insert into a lipid membrane, these peptides rely on a combination of membrane electrostatic and hydrophobic interactions with lipid-acyl chains and consequently there may be less selectivity for bacterial membranes.^15,16^ This lack of selectively can limit applications due to increased cytotoxicity toward mammalian cells. Peptides with β-sheet structures generally show less efficiency with membrane insertion but are more selective toward negatively charged membranes than neutral mammalian membranes.^17^ In nature, this conformation is stabilized by the presence of disulfide bridges between the side chains. Synthetic AMPs free of cysteine residues have been designed to facilitate hairpin folding at the membrane interface leading to membrane disruption and two AMPs with such a hairpin structure, BTT2-4 and BTT6, displayed Gram-negative selectivity and low cytotoxicity.^18^

Here, we aim to identify novel analogues of A3a with increased Gram-negative activity and selectivity over mammalian cells by creating analogues of A3a designed to stabilize its hairpin structure on the basis that molecular dynamic (MD) simulations show that the hairpin structure of both A3 and A3a might be more stable in the more active amidated analogue (Table 1). Of three new analogues, we find one to be almost completely inactive, but two analogues have several features that positively distinguish them from the parent A3a including the ability to provide effective therapy in a *Galleria mellonella* burn wound *A. baumannii* infection model.

**Table 1:** A3a and Derivatives along with the Sequences, Rationalizations, and Characteristics of Each Peptide^a^

modification	sequence	rationale	length (aa^b^)	charge	octanol Δ*G*_woct_ (kcal/mol)	octanol-interface Δ*G*_woct_ – Δ*G*_wif_	molecular weight (g/mol)
AamAP1	FLFSLIPHAI GGLISAFK	original peptide from which A3 was derived	18	+1	–3.77	–0.07	1931.32
A3a	FLFSLIRKAI GGLISAFK-NH_2_	increase membrane interaction, stabilize secondary structure	18	+4	–2.59	0.11	1980.44
A3a[R7K]	FLFSLI**K**KAI GGLISAFK-NH_2_	stabilize secondary structure, decrease cytotoxicity	18	+4	–2.41	0.16	1952.43
A3a[I(6,10)-del]	FLFSL_RKA_ GGLISAFK-NH_2_	decrease production cost, promote AGGA loop formation, decrease cytotoxicity	16	+4	–1.97	0.23	1754.13
A3a[I14W]	FLFSLIRKAI GGL**W**SAFK-NH_2_	increase β-sheet stability, increase insertion	18	+4	–4.13	0.14	2053.49

a Molecular weights were calculated using peptide2.com.

b Amino acid; octanol and octanol-interface whole residue hydrophobicity scales calculated according to Wimley–White.^19^

## Results and Discussion

### MD Simulations of A3 and A3a

In a previous study by van der Walt et al.,^14^ we determined that the increased positive charge of A3a compared to A3 contributes to higher affinity toward bacterial membranes, due to their overall negative charge. This effect from amidation has also been observed in previous studies.^20,21^ To design analogues of A3a with increased Gram-negative activity and increased selectivity over mammalian cells, all atom MD simulations were used to identify which further structural features of A3a, when compared to A3, might contribute to increased Gram-negative activity.

**Figure 1:**
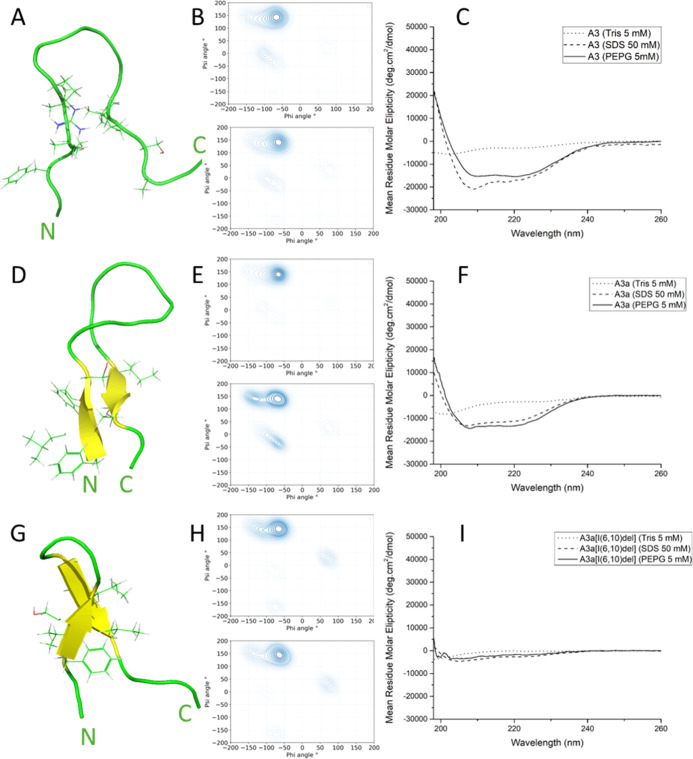
Effect of amidation and truncation on the conformation of A3 when binding to POPE/POPG lipid bilayers. Representative snapshots of one peptide from each of the duplicate MD simulations (A,D,G), Ramachandran plots for the last 20 ns of duplicate 200 ns simulations averaged over four peptides (B,E,H), and far-UV CD spectra (C,F,I) for A3 (A–C), A3a (D–F), and A3a[I(6,10)-del] (G–I). CD spectra were obtained in 5 mM Tris buffer, pH 7, 50 mM sodium dodecyl sulfate (SDS), or 5 mM POPE/POPG (3:1 ratio) small unilamellar vesicles (SUVs). All peptides were prepared to a final concentration of 50 μM.

In the previous work using the same MD simulation methodology, we have identified α-helix conformation for temporin L and a mixture of α-helix and polyproline II for pleurocidin and other peptides from the Winter Flounder.^22−24^ Here, although CD spectroscopy indicates that both A3 and A3a adopt ordered secondary structures, with a spectral characteristic of α-helix conformation when interacting with either anionic detergent or simple models of the Gram-negative model membrane (Figure 1C,F), MD simulations instead characterize peptide–bilayer interactions during the initial binding and penetration of the interface and reveal a variety of hairpin conformations that are adopted in this phase (Figures 1, S1, and S2). The clear difference in the secondary structure between CD and MD results is expected due to the difference in time scale between the two methodologies. CD is measured over minutes and determines the secondary structure of the peptide achieved after minutes, whereas MD measures change in secondary structure upon initial interaction with bilayer environments. In MD simulations of four peptides binding to bilayers comprising 192 zwitterionic POPE and 64 anionic POPG lipids, both A3 and A3a adopt a hairpin conformation (Figure 1A,D). The majority of dihedral angles (φ −75°, ψ 150°) in both peptides are consistent with polyproline-II (P_II_) conformations^25^ (Figure 1B,E). However, Ramachandran angles consistent with other conformations are also observed, including type-I β-turn (φi + 1 −60°, ψi + 1 −30°, φi + 2 −90°, ψi + 2 0°) and antiparallel β-sheet (φ −140°, ψ 135°) (Figure 1B,E). The β-sheet conformation is most evident, in one replicate simulation only, for A3a and this sheet is suggested to be stabilizing the hairpin conformation (Figure 1D). This hairpin is stabilized by phenylalanine residues at positions 1, 3, and 17; serine residues at 4 and 14; and lysine residues at 8 and 18.^26^

### Rational Design and MD Simulation Analyses of A3a Analogues

The main aim of this study is to increase the activity of A3a against Gram-negative pathogens. Since both A3 and A3a adopt a hairpin structure with a zipper between the two termini separated by a relatively disordered loop forming in the central region of the peptide, the aim was to design A3a analogues that further increase the stability of the secondary structure to test whether indeed a more stable hairpin conformation promotes enhanced antibacterial potency.

Espinosa et al.^27^ showed that certain amino acids, such as tryptophan and lysine, promote the stabilization of hairpin structures. Li et al.^28^ concluded that the mode of action of AMPs is strongly related to the secondary structures of AMPs. Small modifications of the structure may result in changes in the biophysical properties and activity. Keeping this in mind, further modifications through substitution, deletion, and repositioning of certain residues were made to improve A3a (Table 1). This resulted in the creation of three different analogues, namely, A3a[R7K], A3a[I(6,10)-del], and A3a[I14W].

For the first analogue, A3a[R7K], arginine 7 was replaced with lysine to promote the stabilization of the hairpin structure.^27^ Some studies have also suggested that substitution of arginine with lysine can reduce the toxicity of AMPs by reducing the potential of hydrogen bonds forming with zwitterion lipids.^29^

In order to increase the amphipathicity and subsequent membrane insertion of A3a,^30,31^ isoleucine 14 was substituted with tryptophan to create the analogue A3a[I14W]. Previous studies have shown that the addition of tryptophan generally increases the antimicrobial activity of AMPs,^32^ and tryptophan can further stabilize the hairpin structure within peptides.^27^

The MD simulations show that the central loop of A3a is relatively disordered and that Ile6 and Ile10 are not interacting with or inserting into the bilayer (Figure S2B). Therefore, the final analogue A3a[I(6,10)-del] was designed to promote loop formation within the center of the peptide by eliminating steric hindrance caused by the Ile6 and Ile10 side chains. Further advantages include the potential reduction in cytotoxicity of A3a with the removal of these isoleucine residues by increasing amphipathicity,^33^ as well as the reduction in synthesis costs.

### Outcome of Rational Design—Antibacterial Potency

A3a and its designed analogues were screened against a panel of ESKAPE pathogens (Table 2), and a minimum of a 4-fold change in potency is commonly considered a significant difference. The activity of A3a against this panel differs to that of previous studies,^14^ most likely due to the differences in the bacterial strains and methodologies used, in particular, the use of cation-adjusted Mueller Hinton broth (MHB). Nevertheless, clear differences in antibacterial potency can be attributed to the changes made to the A3a sequence. While A3a[I(6,10)-del] has no antibacterial activity, A3a and the other two analogues have potent activity against Gram-positive *S. aureus* (MSSA) ATCC 25923. Against a panel of Gram-negative bacterial pathogens, the activity of A3a[R7K] is broadly comparable to that of its parent A3a, modestly but significantly greater only against *E. coli* ATCC 25922. In contrast, the antibacterial activity of A3a[I14W] is significantly greater than that of the parent A3a against all Gram-negative isolates tested. Previously, we have used a QSAR approach to consider factors that determine potency in a large panel of AMPs and both hydrophobicity and lipophilicity correlate strongly with anti-Gram-negative activity and others have also focused on lipophilicity.^14,34^ Here, although A3a[I14W] may have a greater propensity to partition into octanol, taken as a proxy for the hydrocarbon core of a bilayer and hence lipophilicity, considering whole residue hydropathy using a combination of octanol and interface hydrophobicity scales indicates there is little difference between A3a[I14W], A3a[R7K], and A3a. Both A3a[R7K] and A3a[I14W] have potentially useful activity against *A. baumannii* ATCC 19606, which is also the only Gram-negative pathogen sensitive to A3a.

**Table 2:** MICs (μg/mL) of A3a and Analogues against ESKAPE Pathogens^a^

	E. coli ATCC 25922	*A. baumannii* ATCC 19606	K. pneumoniae ATCC BAA-1705	E. cloacae ATCC 700323	P. aeruginosa ATCC 27853	S. aureus (MSSA) ATCC 25923
A3a	>256	32 [16]	>256	>256	>256	8 [4]
A3a[R7K]	**128 [65]**	16 [8]	>128	>128	>128	8 [4]
A3a[I(6-10)-del]	>128	**>128**	>128	>128	>128	**>128**
A3a[I14W]	**32 [16]**	**8 [4]**	**128 [62]**	**128 [62]**	**128 [62]**	4 [2]
**controls**						
ciprofloxacin	0.25 [0.76]	1 [3]	>32	0.125 [0.377]	0.5 [1.5]	0.5 [1.5]
gentamicin	0.5 [1.0]	32 [67]	2 [4]	2 [4]	4 [8]	0.5 [1.0]
polymyxin B	0.5 [0.4]	1 [0.76]	0.5 [0.4]	0.25 [0.19]	2 [1.5]	>32

a (>) indicates no activity observed at the highest concentrations tested. Values in brackets are MIC values as μM. Values in bold indicate a significant (≥4-fold) change in MIC relative to A3a. The MIC was defined as the lowest concentration that resulted in pathogen growth of <0.1 above the background absorbance.

### Deletion of Ile6 and Ile10 Restricts Conformational Flexibility and Disrupts the A3a Secondary Structure

Although CD spectra obtained for A3a[R7K] and A3a[I14W] are very similar to those obtained for the parent A3a (or A3) peptides (Figure S1C,F), the spectra obtained for A3a[I(6,10)-del] indicate that this peptide alone is unable to adopt the ordered α-helix conformation in the steady state. The steady state here refers to the conformation adopted by the peptide at the equilibrium position obtained after 10 s of minutes when samples are prepared and acclimatize to the conditions in the CD spectrometer. The deletion of two isoleucine residues compromises its ability to adopt the same conformations as the parent peptide (Figure 1I). This indicates that the secondary structure of A3a is heavily influenced by either length or the lipophilicity conferred by the presence of Ile6 and Ile10 (Table 1).

**Figure 2:**
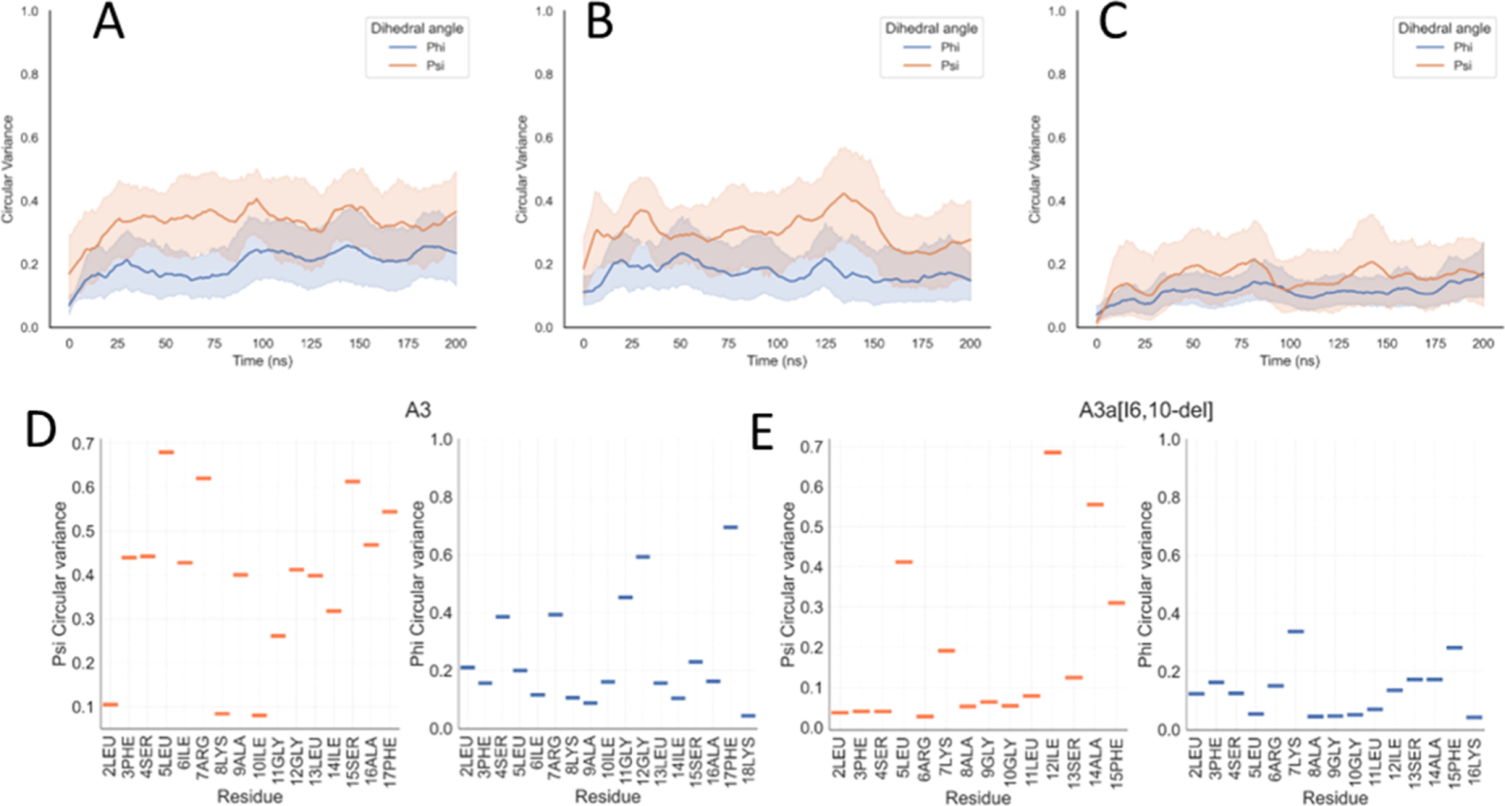
Deletion of Ile6 and Ile10 attenuates conformational flexibility when binding to POPE/POPG lipid bilayers. Circular variance for φ and ψ Ramachandran angles averaged over four peptides over the 200 ns duration (A–C) or time averaged by amino acid residue (D,E), representative of duplicate MD simulations for A3 (A,D), A3a (B), and A3a[I(6,10)-del] (C,E).

MD simulations also indicate substantially altered conformational behavior for A3a[I(6,10)-del] relative to the parent peptides and the other new analogues. Interestingly, however, this difference is revealed most notably in the recorded Ramachandran angle circular variance, rather than the conformation itself. Calculating the circular variance of dihedral angles for peptides throughout the MD simulations enables the determination of the conformational flexibility of peptide secondary structures,^35^ and in the present simulations, it will be affected by any switch between polyproline-II, β-turn, or β-sheet conformations over the duration of the simulation. Conformational flexibility, as revealed by low circular variance, is reproducibly and substantially restricted in A3a[I(6,10)-del] (Figures 2C and S3C), for both ψ and φ, relative to that observed for A3 or A3a (Figures 2A,B and S3A,B). This may be interpreted as elimination of the ability of many residues in the peptide to adopt differing conformations as the bilayer is penetrated, with the impact being felt most by residues at the N-terminus and between Ala8 and Leu11 (Figure 2D,E).

In MD simulations, there are few substantial changes in conformational behavior observed for the remaining analogues (Figures S1 and S4). Although substantial β-sheet is observed for A3a[I14W] in one replicate simulation, this is again not reproduced in the duplicate simulation (Figure S1D,E). No substantial changes are observed in conformational flexibility either, as reported on by circular variance (Figure S4).

The loss of both conformational flexibility in the MD simulations and the steady-state α-helix conformation is therefore unique for A3a[I(6,10)-del], among the five peptides tested, and the two properties are likely related. Further, since antibacterial activity is completely lost for A3a[I(6,10)-del] (Table 2), these properties are also likely essential for effective antibacterial activity.

The relative importance of conformational flexibility for the antibacterial potency of different AMPs is perhaps underappreciated. For temporin L, an AMP from the frog *Rana temporaria* which has greater antibacterial potency than A3a, the first nine residues form an ordered α-helix with almost zero circular variance around the ψ or φ dihedral angle and conformational flexibility is restricted to the four residues at the C-terminus.^22^ In contrast, a similar analysis of pleurocidin, a very potent AMP from Winter Flounder, finds that this peptide can adopt both α-helix and P_II_ conformations and that increasing conformational flexibility is found in the lysine-to-arginine-substituted analogue which has greater antibacterial potency.^23^ As such, high conformational flexibility can be a feature of, or largely absent from, potent AMPs. For A3a and its analogues, some conformational flexibility is required for antibacterial activity, and modifications that eliminate this property are unlikely to be tolerated.

We focus now on understanding and characterizing in more depth the two more active analogues, in particular, A3a[I14W], which may have potential for further development.

### MD Simulations Do Not Detect Substantial Changes in Lipid Bilayer Binding

Since many AMPs are thought to exert their antibacterial action through damaging or crossing the bacterial plasma membrane, we asked if MD simulations can reveal whether binding, penetration, and/or peptide aggregation in the bilayer are affected in the two more active A3a analogues.

Residues involved in hydrogen bonding with lipid head groups are the same between A3a analogues (Figure S5). These residues include those with positive net charges, notably the N-terminal Phe1 which has a free primary amino group and the three residues with cationic side chains: Arg/Lys7; Lys8; and Lys18. The two serine residues also contribute to peptide–lipid hydrogen bonding. Weak hydrogen bonding in the C-terminal segment (Ala9–Lys18) of A3 appears attenuated in A3a (Figure S5A,B), and this may be restored in A3a[R7K] and A3a[I14W] but the effect is inconsistent and observed in only one replicate simulation for each peptide (Figure S5C,D).

Penetration of the peptides into the bilayer is also similar, although a trend to greater penetration by A3a[I14W] may be detected (Figure S6). Calculating the center of mass of each peptide relative to the bilayer center allows a measure of penetration over time, with substantial differences often observed according to peptide or lipid bilayer composition.^24^ A3a[I14W] is the only one of the active A3a analogues to penetrate deeper in the last 50 ns than over the first 100 ns of the simulation (*p* = 0.0429) (Figure S6B). This analysis is however compromised to an extent by the more heterogeneous penetration observed for A3a and A3a[R7K] and also the phenomenon whereby at least one A3a[R7K] and A3a[I14W] peptide crosses the periodic boundary conditions and hence is excluded from the penetration analysis. This phenomenon is reproduced in both replicates for each of these analogues and is not observed for parent A3a (or A3).

Depending on the mechanism of action of a peptide, aggregation can have either positive or negative effects on antibacterial activity.^36,37^ Consistent aggregation between specific residues of peptides or proteins is characteristic of certain mechanisms of membrane permeabilization, such as with pore-forming peptides.^38^ Inconsistent and erratic aggregation can, however, interfere with peptide activity, preoccupying residues important for membrane interaction or insertion. Evaluation of the degrees of aggregation between individual peptides, and the residues involved, showed modifications could be made to increase or decrease aggregation. Evaluation of peptide aggregation reveals that, although both A3 and A3a consistently exist as tetramers for the majority of both replicate simulations, larger segments of A3a are involved in aggregation when compared with A3 (Figure S7A–C). Both A3a[R7K] and A3a[I14W] exist in lower-order aggregates relative to A3a (Figure S7A,D,E), but again this analysis is compromised by the tendency of these two peptides to cross the periodic boundary conditions and hence be unavailable for aggregation. While the penetration and aggregation behavior of A3a and its analogues may be further explored in future, for the present study, we now focus on establishing differences in behavior in vitro.

### A3a[R7K] Quickly Permeabilizes *A. baumannii* ATCC 19606 but Both A3a[R7K] and A3a[I14W] Are Rapidly Bactericidal

**Figure 3:**
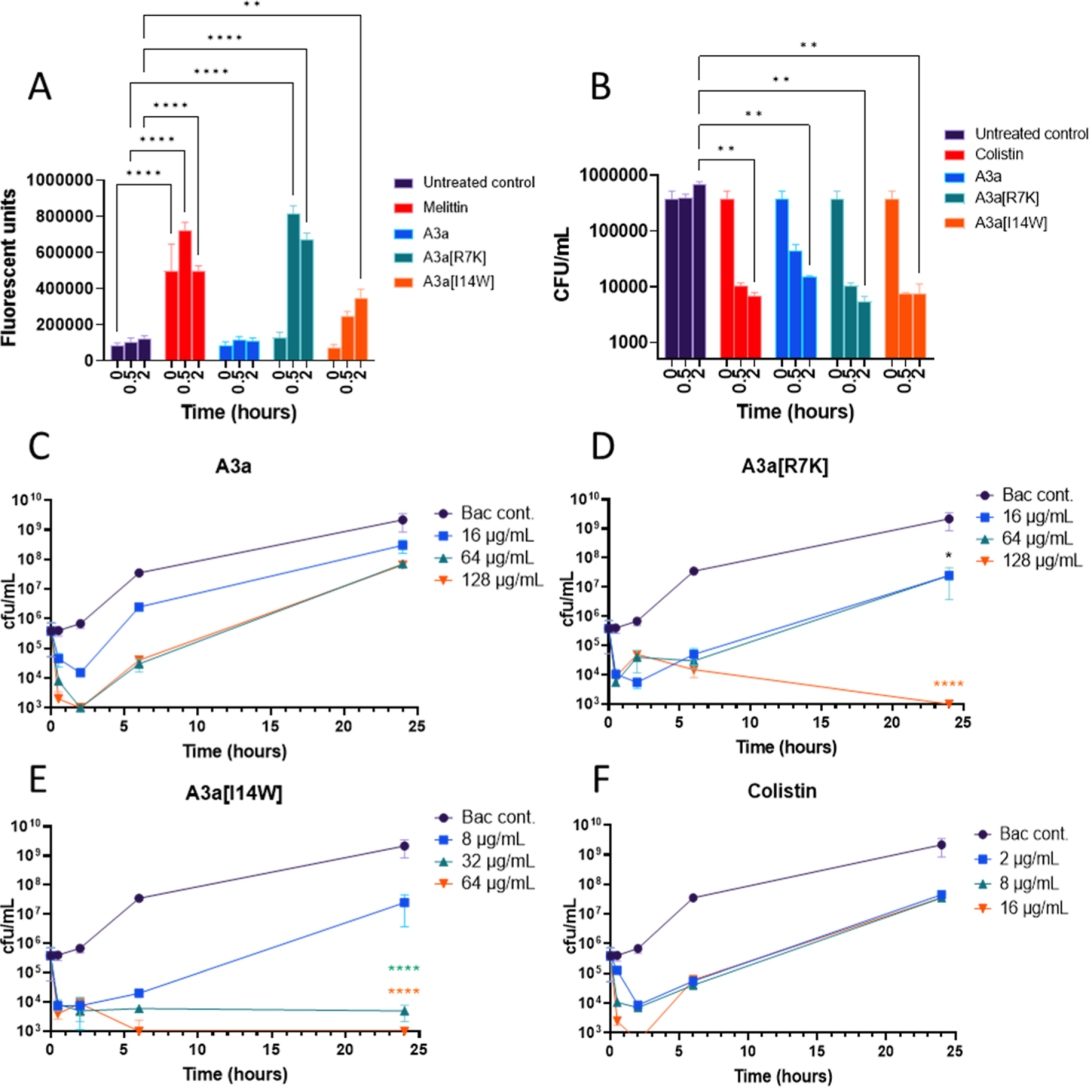
A3a and its analogues are all rapidly bactericidal but only A3a[R7K] permeabilizes *A. baumannii* ATCC 19606. Membrane permeabilization of *A. baumannii* ATCC 19606 by A3a, A3a[R7K], and A3a[I14W] was determined at 1× MIC values after 0.5 and 2 h exposure with the SYTOX Green assay (A). Killing kinetics was performed in the same conditions (B). Killing kinetics are also shown for A3a (C), A3a[R7K] (D), A3a[I14W] (E), and colistin (F) over 24 h. A3a[R7K] and A3a[I14W] were tested at 1×, 4×, and 8× their MIC values. The MIC value of A3a was too high to be tested using this method and was therefore tested at the same concentration as A3a[R7K]. Two-way analysis of variance (ANOVA) with Dunnett’s multiple comparison test indicates significant difference between untreated controls and peptide treatments at each time point (*p < 0.05, **p < 0.005, ****p < 0.0001). Data is representative of three independent biological repeats.

To determine if bacterial killing is associated with membrane permeabilization, the SYTOX Green assay was used to compare permeabilization between A3a and its two more potent analogues. The control, melittin, causes rapid membrane permeabilization (Figure 3A). In contrast, no membrane permeabilization is observed for A3a after 2 h of exposure. A3a[I14W] causes significant membrane permeabilization after 2 h but not after 30 min, and the extent of permeabilization induced is much less than that induced by A3a[R7K] which is both substantial and significant after 30 min of exposure. The mechanistic reasons for increased membrane permeabilization with A3a[R7K] are as yet unclear.

There is a close relationship between peptide secondary structure and the ability to permeabilize membranes. Eiríksdóttir et al.^39^ evaluated the secondary structure of cell-penetrating peptides (CPPs) and reported that CPPs that adopt a β-sheet structure are more sensitive to membrane charge than α-helical peptides. For the latter, a combination of electrostatic and hydrophobic interactions is required, while for β-sheet structures, interaction with negatively charged membranes is required for the stabilization of the secondary structure. Even though A3a[I14W] does not form a defined β-sheet structure, the hairpin conformation being adopted closely resembles the hairpin β-sheet structure described by Eiríksdóttir et al.^39^ In the presence of cells with zwitterionic membranes, such as HaCat cells, A3[I14W] could be less likely to adopt this hairpin structure, accounting for the increased selectivity of A3[I14W].

A3a[I14W] causes no significant membrane permeabilization after 30 min of treatment, even though cell death is already present. Other studies have found that AMPs with hairpin structures also target LPS and DNA. The low amount of membrane permeabilization caused by A3a[I14W] indicates that this AMP could target either outer membrane-associated LPS or has intracellular targets such as DNA. Tram et al.^18^ showed that peptides designed to adopt a β-hairpin structure have disordered structures in an aqueous environment but adopt β-sheeted structures when in the presence of LPS, indicating that interacting with LPS actually promoted the formation of these peptide secondary structures. Powers and Hancock^17^ identified that peptides with hairpin β-sheet structures, such as tachyplesins, have higher affinity for LPS compared with AMPs consisting of more extended structures, such as indolicidin.

Tachyplesins also have intracellular targets, binding to the minor groove of double-stranded DNA.^40^ A similar property may account for A3a[I14W] having better antibacterial activity compared with A3a[R7K], despite permeabilizing membranes to a lesser extent.

As A3a[R7K] and A3a[I14W] have good selective activity against *A. baumannii* ATCC 19606, further mode-of-action studies were undertaken using this pathogen. Rapid killing is associated with the disruption of membrane integrity that leads to cell death.^41^ At the MIC of A3a, A3a[R7K], and A3a[I14W], the colony forming unit (CFU)/mL is reduced 3× log-fold after 30 min, similar to colistin (Figure 3B). However, the reduction in CFU/mL is significant only after 2 h compared with the untreated control.

To determine whether the peptides were bacteriostatic or bactericidal, killing kinetic studies were undertaken for 24 h against *A. baumannii* ATCC 19606 at 1×, 4×, and 8× the MIC (Figure 3C–F). The positive control for this experiment, colistin, shows regrowth at all of the concentrations tested.

Previous studies have shown the same effects when tested against different strains of *A. baumannii*, including ATCC 19606. This regrowth of cells after treatment has aided in the rise of resistance against colistin and other antibiotics.^42^ A3a shows regrowth at 1×, 4×, and 8× MIC after 24 h. In contrast, A3a[R7K] prevents regrowth at 8× MIC and A3a[I14W] prevents regrowth at 4× and 8× MIC. This shows that the bactericidal effects of A3a[R7K] and A3a[14-W] are concentration dependent, and at lower concentrations, bacterial proteolytic enzymes may degrade the AMPs and a higher effective dosage is required for complete killing. Fosgerau and Hoffmann^43^ highlight the importance of increasing the half-life of peptides through methods that limit enzymatic degradation or bind to plasma albumin and/or other proteins.

### A3a[R7K] and A3a[I14W] Have Improved Selectivity Indexes

**Figure 4:**
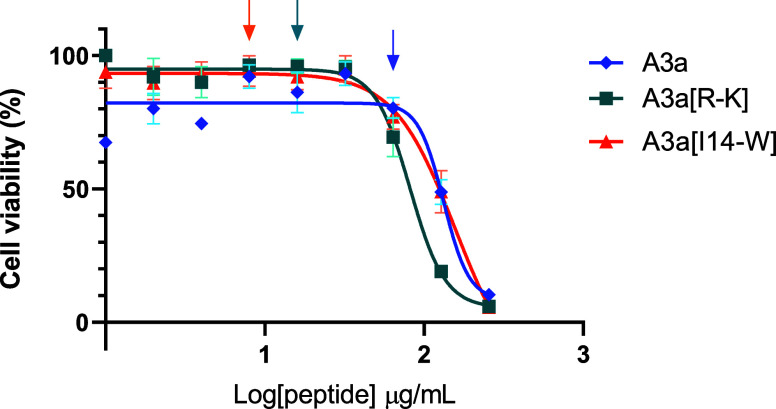
Cytotoxicity of A3a, A3a[R7K], and A3a[I14W] against HaCat cells. Cell viability was measured with the MTT assay after 24 h exposure at 1–256 μg/mL. The IC_50_ value of A3a was 118.3 ± 15.75 μg/mL, and for A3a[R7K] and A3a[I14W], it was 82.6 ± 9.05 μg/mL and 114.5 ± 18.15 μg/mL, respectively. Arrows indicate the MIC values of peptides against *A. baumannii* ATCC 19606 correlating to colors of the different curves. Data is representative of three independent biological repeats.

A high selectivity index is required to further develop AMPs for therapeutic purposes, and therefore, cytotoxicity was evaluated using the HaCat cell line. In an initial screening at 256 μg/mL, cytotoxicity was observed for A3a, A3a[R7K], and A3a[I14W]. The IC_50_ for A3a, A3a[R7K], and A3a[I14W] against HaCat cells is 118.3 ± 15.75, 82.6 ± 9.05 μg/mL, and 114.5 ± 18.15 μg/mL, respectively (Figure 4). Two-way ANOVA shows that there is a significant increase in cytotoxicity from A3a to A3a[R7K] (*p* < 0.05) but not from A3a to A3a[I14W]. Both new analogues therefore show higher selectivity toward *A. baumannii* ATCC 19606. The ratio of HaCat IC_50_ to *A. baumannii* ATCC 196060 MIC is only 3.70 for A3a. This increases slightly to 5.16 for A3a[R7K], but for A3a[I14W], it increases to 14.31, a nearly 4-fold improvement when compared with A3a.

### Despite In Vivo Toxicity, Both A3a[R7K] and A3a[I14W] Provide Therapeutic Protection in an Insect Burn Wound Infection Model

Finally, we evaluated the efficacy of A3a, A3a[R7K], and A3a[I14W] in a *G. mellonella* in vivo burn model. *G. mellonella* larvae have innate immune systems similar to that found in humans.^44^ This allows for a low-cost, high-throughput in vivo model for testing potential antibiotic treatments on larvae infected with the pathogen of choice. Maslova et al.^45^ developed a topical variation of this methodology, in which larvae are lightly burned, after which the burn wounds are infected and then treated with the antibiotic in question. This model has proven effective in identifying the benefits of synergy between AMPs in enabling effective therapy of burn wounds infected with *A. baumannii*.^24^

**Figure 5:**
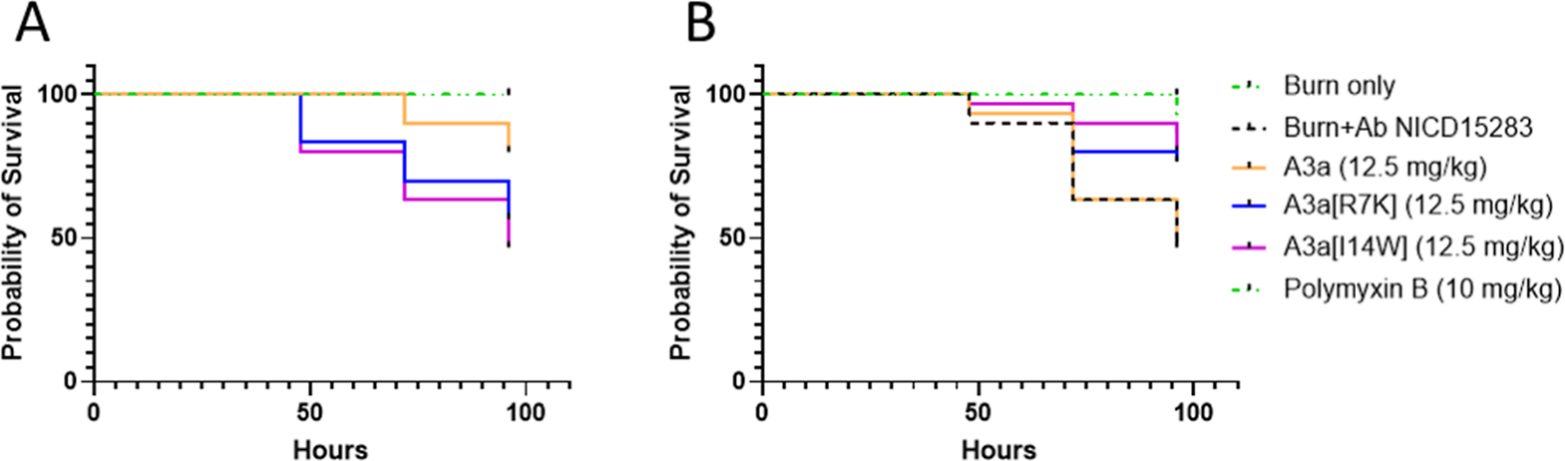
New analogues of A3a have therapeutic potential despite in vivo toxicity. Toxicity (A) and therapy post *A. baumannii* NICD 15283 infection (B) in a *G. mellonella* burn wound model. The indicated doses of A3a, A3a[R7K], A3a[I14W], or polymyxin B were applied to larvae with either burns only (A) or *A. baumannii* NICD 15283-infected burns (B). Despite toxicity associated with both A3a analogues, they both mitigate the effects of infection.

In the present study, burn alone does not affect larvae survival, with 100% of the larvae surviving for 96 h post injury (Figure 5A). However, mortality associated with AMP toxicity is observed in the absence of infection. At a dose of 12.5 mg/kg, A3a causes significant mortality (*p* = 0.0104) to burnt but uninfected larvae, reducing survival to 80%. At the same dose, however, both A3a[R7K] and A3a[I14W] have substantial and significantly (*p* < 0.0001) reduced survival of 56.7% and 46.7%, respectively (Figure 5A). All three peptides therefore are toxic toward the uninfected larvae, but this is more pronounced for A3a[R7K] and A3a[I14W].

Infection with *A. baumannii* NICD 15283 also causes substantial and significant mortality (*p* < 0.0001) with only 46.7% surviving after 96 h (Figure 5B). At the same dose of 12.5 mg/kg where A3a has toxicity, it offers no significant improvement in survival for infected larvae (*p* = 0.7879) with only 50% of larvae surviving. In contrast, however, and despite their higher toxicity, both A3a[R7K] (*p* = 0.0245) and A3a[I14W] (*p* = 0.0066) peptides offer significant protection from the *A. baumannii* NICD 15283 infection, increasing survival to 76.7 and 80%, respectively.

This is a clear improvement over that of the parent peptide, A3a, which offers no significant protection at 12.5 mg/kg. The decreased toxicity in the presence of bacterial infections indicates that the peptides have a selectivity toward bacteria over the larval cells when both are present. Therefore, although the toxicity in uninfected wounds will need to be addressed in future studies, survival rates in infected larvae show promise for the further development of these peptides as therapeutic agents. Since peptides are likely proteolytically degraded by *A. baumannii* before affecting larvae negatively, there is scope in the future for using dose–response experiments to determine whether therapy can be achieved at lower doses while reducing risk from toxicity. Additionally, a focus on increasing peptide charge and interspacing hydrophobic residues with polar residues has been effective in reduce toxicity,^11,32^ while other approaches such as the lipid envelopment of peptides,^46^ conjugation of AMPs to nanoparticles,^47−49^ and cyclization of AMPs^50,51^ may be of use.

Overall, both A3a[R7K] and A3a[I14W] show some promise for their therapeutic use. For A3a[R7K], its potential may be limited by the finding that, while it effectively inhibits *A. baumannii* ATCC 19606 through membrane permeabilization, at concentrations 16× lower than the minimum lethal dose (MLD) against HaCat cells, it is only bactericidal at 8× MIC. In contrast, A3a[14-W] has broader spectrum activity against the ESKAPE pathogens, with higher selectivity toward *A. baumannii*, and is rapidly bactericidal despite decreased membrane permeabilization. Taken together, this suggests a substantially different mechanism of action for A3a[I14W], requiring further identification of other cellular targets.

## Conclusions

Through rational design and MD simulations, three analogues of A3a were designed with the aim of increasing the stability of the hairpin structure of A3a, thereby potentially increasing activity and selectivity. The evaluation of antibacterial activity against a panel of ESKAPE pathogens identified increased activity for two analogues, namely, A3a[R7K] and A3a[I14W]. More specifically, A3a[I14W] is active against all Gram-negative pathogens, while A3a[R7K] shows activity against *E. coli* and *A. baumannii.* Both rapidly kill *A. baumannii* ATCC 19606, with A3a[R7K] showing greater membrane permeabilization. In contrast, although A3a[I14W] is less membrane active, this AMP is more bactericidal, indicating a different mode of action for A3a[I14W]. In vivo studies using *G. mellonella* larvae show that both peptides provide significant protection against *A. baumannii* infections, despite being somewhat toxic in the absence of an infection. Both A3a[R7K] and A3a[I14W] are novel peptides with improved selectivity for Gram-negative pathogens, with different mechanisms of action highlighting again that small changes in peptide sequence can lead to substantial changes in behavior. Finally, the use of MD simulations and analysis helps to understand the effectiveness of peptide modifications in the pursuit of developing improved treatments against MDR pathogens.

## Materials and Methods

### MD Simulations

To determine how the different peptides interact with bacterial cell membranes, the peptides were placed in close proximity to POPE/POPG lipid bilayers that simulate Gram-negative cell membranes in silico as described by Manzo et al., 2020.^23^

For all peptides, the starting structures within each simulation (Figure S8) were obtained by inserting a single peptide with an extended structure into a water box containing 0.15 M NaCl and allowing the peptides to adopt a secondary structure within a 1 μs simulation. The final conformations of these peptides were then used within membrane simulations.

All bilayers in this study contained 256 lipids (128 lipids per layer). Gram-negative membranes were simulated by lipid bilayers that consisted of 1-palmitoyl-2-oleoyl-*sn*-glycero-3-phosphoethanolamine (POPE) and 1-palmitoyl-2-oleoyl-*sn*-glycero-3-phosphoglycerol (POPG) with a 75:25 (POPE/POPG) molar ratio as used in previous studies.^52,53^ Four peptides were placed approximately 3 nm above the lipid bilayer and randomly arranged at least 2 nm apart. The system was solvated by adding TIP3P water,^54^ Cl^–^-, and Na^+^-ions to neutralize the system. The final salt concentration was 0.15 M to approximate the physiological systems. Energy minimization was carried out at 310 K with the Nose–Hoover thermostat using the steepest descent algorithm until the maximum force was less than 1000.0 kJ/mL/nm (∼3000–4000 steps). Equilibration was carried out using the *NVT* (constant number of particles, volume, and temperature) ensemble for 100 ps and then the *NPT* (constant number of particles, pressure, and temperature) ensemble for 1 ns with positional restraints on the peptides. For this, a Berendsen thermostat and barostat were used. Hydrogen-containing bond angles were constrained with the LINCS algorithm. Final simulations were run in the *NPT* ensemble using 2 fs intervals, with trajectories recorded every 2 ps. This was done using a Nose–Hoover thermostat and a Parrinello–Rahman barostat. Force fields for atoms were calculated using CHARMM36.^55^ All simulations were run for a total of 200 ns and repeated twice with peptides inserted at different positions and orientations.

The data from the simulations was then used to determine the number of residues contributing to membrane insertion, the region of the peptide inserting, the residues contributing to hydrogen bonding, the aggregation pattern, size, and stability, the associated secondary structure, and circular variance. This was done using MD Analysis.^56^ Bilayer insertion was determined by measuring the *Z*-positions of amino acid α-carbons relative to the average *Z*-positions of lipid phosphate groups. Hydrogen bonding was determined by looking at atoms within peptides capable of forming hydrogen bonds with lipid head groups and measuring the distance between these atoms. Distances ranging between 4 and 6 Å were considered to be hydrogen bonds. This same method was used for aggregation patterns, but the atoms in question were between individual peptides instead. Secondary structures of peptides were measured by looking at φ- and ψ-angles of the peptide backbones. Circular variance was determined by calculating the changes in these angles throughout the simulations.

### Peptide Synthesis and Purification

All peptides used in this study were purchased from GenScript (Piscataway, New Jersey, USA). The purity (>95%) and molecular mass of peptides were determined by the vendor using reverse-phase HPLC and mass spectrometry, respectively. Peptides were freeze-dried and stored at −80 °C until needed.

### Circular Dichroism Spectroscopy

Far-UV CD spectra of the peptides were obtained in SDS micelles (50 mM made up in 5 mM Tris, pH 7.4) and in the presence of SUVs using a J-810 spectropolarimeter (Jasco, Johannesburg, South Africa). To prepare the SUVs, lipid powders were solubilized in chloroform and dried under rotor-evaporation. To completely remove the organic solvent, the lipid films were left overnight under a vacuum and hydrated in 5 mM Tris buffer (pH 7.4). The lipid suspension was subjected to five rapid freeze–thaw cycles for further sample homogenization. POPE/POPG (75:25, mol/mol) SUVs were obtained by sonicating the lipid suspensions on Soniprep 150 (Measuring and Scientific Equipment, London, UK) for 2 × 5 min with an amplitude of six microns in the presence of ice to avoid lipid degradation. The SUVs were stored at 4 °C and used within 5 days of preparation. Far-UV CD spectra were recorded from 260 to 180 nm at a constant temperature of 296.15 K, with a bandwidth of 2 nm, a step size of 1 nm, and a path length of 0.5 mm. The POPE/POPG SUV suspensions at a final concentration of 5 mM were used to dissolve the peptides to yield a final peptide concentration of 50 μM. The same experimental conditions were used to evaluate the peptide secondary structure in 50 mM SDS micelles. For data processing, a spectrum of the peptide-free SDS or lipid suspension was subtracted and Savitsky–Golay smoothing with a convolution width of 4 points was applied.

### Antimicrobial Activity

Antimicrobial activity was determined against a panel of ESKAPE pathogens. Briefly, streak plates were grown for *E. coli* ATCC 25922, *S. aureus* ATCC 25923, *K. pneumoniae* ATCC BAA-1705, *A. baumannii* ATCC 19606, *P. aeruginosa* ATCC 27853, and *Enterobacter cloacae* ATCC 700323 on tryptic soy agar plates from frozen glycerol stocks. Bacterial colonies were picked and cultured in cation-adjusted MHB at 37 °C with 180 rpm shaking. Cultures were back-diluted to reach a starting OD_600_ of 0.1 and then diluted 100-fold to approximately 1 × 10^6^ CFU/mL.

The peptides were dissolved in MHB, serially diluted (2 to 128 μg/mL), and added to 96-well polypropylene plates along with the different pathogens at a 1:1 ratio. Polymyxin B, gentamicin, and ciprofloxacin were used as positive controls at a concentration range of 0.063 to 32 μg/mL. The activity of the peptides was determined by measuring the optical density (600 nm) of cell growth after 20 h of incubation. The MIC was defined as the lowest concentration that resulted in the pathogen growth of <0.1 above the background absorbance.

### Cytotoxicity against Mammalian Cells

HaCat cells were purchased from ECACC and were cultured in Dulbecco’s modified Eagle’s medium (DMEM) supplemented with 10% heat-inactivated fetal calf serum (FCS) and 1% antibiotic–antimycotic (DMEM/FCS). To determine the initial cytotoxicity of all peptides, HaCat cells were plated at 5.56 × 10^4^ cells/mL (90 μL) in a 96-well plate. After an overnight incubation at 5% CO_2_, 37 °C, and 95% humidity, for initial screening peptide at a final concentration of 256 μg/mL (10 μL) were added triplicate wells. For the determination of the IC_50_, of the most active peptides, serial 2-fold dilutions were added. The positive control was 0.1% Triton-X100. After 24 h, cell viability was determined with the 3-(4,5-dimethyl-2-thiazolyl)-2,5-diphenyl-2*H*-tetrazolium bromide (MTT) assay. A volume of 10 μL of a 1 mg/mL MTT was added to each well. After 3 h incubation, the medium was discarded, and the plate was rinsed and dried before the formazan crystals were dissolved by adding 25% DMSO in ethanol. The cell viability was determined by measuring the absorbance at OD_595_. The percentage cell viability was calculated relative to that of the untreated control. The IC_50_ was defined as the peptide concentration that causes 50% inhibition of cell growth. This was calculated from a sigmoidal curve of cell growth (%) over log_10_(peptide concentration). The MLD was defined as the lowest concentration of peptide that showed no statistical difference from the 0.1% Triton-X100 positive control.

### Killing Kinetics

*A. baumannii* ATCC 19606 cells were made up to an OD_600_ of 0.1 (10^6^ cfu/mL) and treated at the MIC of each peptide in a final volume of 1 mL. Colistin (8 μg/mL) was used as a positive control. The effect of 1×, 4×, and 8× the MIC of peptides on the killing of bacteria was undertaken to determine whether that killing was either bacteriostatic or bactericidal after 24 h exposure. Following exposure, 10 μL was used from the individual wells to determine the CFUs with the Miles Misra method.^57^ The limit of detection for this method was 1000 CFUs. This was done at 30 min and 2, 6, and 24 h.

### Membrane Permeabilization

For membrane permeabilization studies, *A. baumannii* ATCC 19606 cells were evaluated after exposure to each peptide by measuring fluorescence. The SYTOX Green assay was undertaken according to Zeng et al.,^58^ with some modifications. Briefly, *A. baumannii* ATCC 19606 was stained with SYTOX Green for 5 min and then treated with each peptide at the 1×, 4×, and 8× MIC for 2 h. Melittin (8 μM) was used as the positive control. The fluorescence was measured every 2 min at excitation and emission wavelengths of 485 and 535 nm, respectively (Spectramax, Multimode detection platform, Molecular Devices, Austria).

### *G. mellonella* Burn Model

Standardized *G. mellonella* larvae were obtained from the FABI Biocontrol center at the University of Pretoria. The larvae were at the life cycle stage not requiring feeding. Prior to use, larvae were sorted into Petri dishes (10 larvae per plate) lined with Whatman filter paper (Fisher, UK) and stored at 28 °C until use. A burn was induced with a heated nail head to achieve a burn wound area of approximately 2 mm^2^. Immediately after burn, the wound was inoculated with a single colony of *A. baumannii* NICD 15283 applied directly to the burn site with an inoculation loop. Peptides were prepared in PBS to final concentrations of 12.5 mg/kg and 15 mg/kg based on the average weight of the group of larvae treated. The peptides were applied topically 1 h after infection by applying a 5 μL drop directly to the burned area. Untreated controls received 5 μL of PBS or polymyxin B (10 mg/kg) instead. Larvae were left at 37 °C and survival was monitored over 96 h. Mortality was recorded as complete melanization of the larval body and complete loss of motility. Three independent experiments were conducted on three different occasions using 10 larvae per treatment/control group (*n* = 30).

### Statistical Analysis

Three biological repeats were performed in triplicate for all assays and results were expressed as the mean ± standard error of the mean. Statistical analysis was performed using GraphPad Prism 10 (San Diego, California, USA). ANOVA was performed followed by a posthoc multiple comparison test. A *p*-value <0.05 was considered statistically significant. Survival curve analysis was by both Logrank (Mantel–Cox test) and Gehan–Breslow–Wilcoxon tests.

## Supporting Information Available

Further analysis of MD simulations and representations of simulated peptide structures (PDF)
