# Supplementary material for: Improving the Activity and Selectivity of a Scorpion-Derived Peptide, A3a, against Acinetobacter baumannii through Rational Design
Source: ACS Omega. 2025 Jan 30;10(5):4699–710. doi: 10.1021/acsomega.4c09593 (PMC11822712; doi:10.1021/acsomega.4c09593)
Supplement: Supplementary file 1 — ao4c09593_si_001.pdf [file ao4c09593_si_001.pdf]

# Improving the activity and selectivity of a scorpion-derived peptide, A3a, against *Acinetobacter baumannii* through rational design

Dalton S. Möller[1], Mandelie van der Walt[1], Carel Oosthuizen[2], Miruna Serian[3,] June C. Serem[4], Christian D. Lorenz[5], A. James Mason[6], Megan J. Bester[4], and Anabella R. M. Gaspar[1]*

[1]Department of Biochemistry, Genetics and Microbiology, Faculty of Natural and Agricultural Sciences, University of Pretoria, Pretoria 0002, South Africa.

[2]Drug Discovery and Development Centre (H3D), University of Cape Town, Rondebosch 7701, South Africa.

[3]Department of Physics, Faculty of Natural, Mathematical and Engineering Sciences, King's College London, WC2R 2LS, UK.

[4]Department of Anatomy, Faculty of Health Sciences, University of Pretoria, Pretoria 0002, South Africa.

[5]Department of Engineering, Faculty of Natural, Mathematical and Engineering Sciences, King's College London, WC2R 2LS, UK.

[6]Institute of Pharmaceutical Science, School of Cancer & Pharmaceutical Science, Faculty of Life Sciences & Medicine, King's College London, SE1 9NH, UK.

*Corresponding authors:

Prof. A. James Mason, Institute of Pharmaceutical Science, King's College London, UK, james.mason@kcl.ac.uk

Prof Anabella R. M. Gaspar, Department of Biochemistry, Genetics and Microbiology, Faculty of Natural and Agricultural Sciences, University of Pretoria, Pretoria, South Africa, anabella.gaspar@up.ac.za

Keywords: scorpion, antimicrobial peptides, rational design, structural characterisation, *Acinetobacter baumannii,* ESKAPE, molecular dynamics simulations, membrane permeabilisation, killing kinetics, *Galleria mellonella*

# Supplementary materials

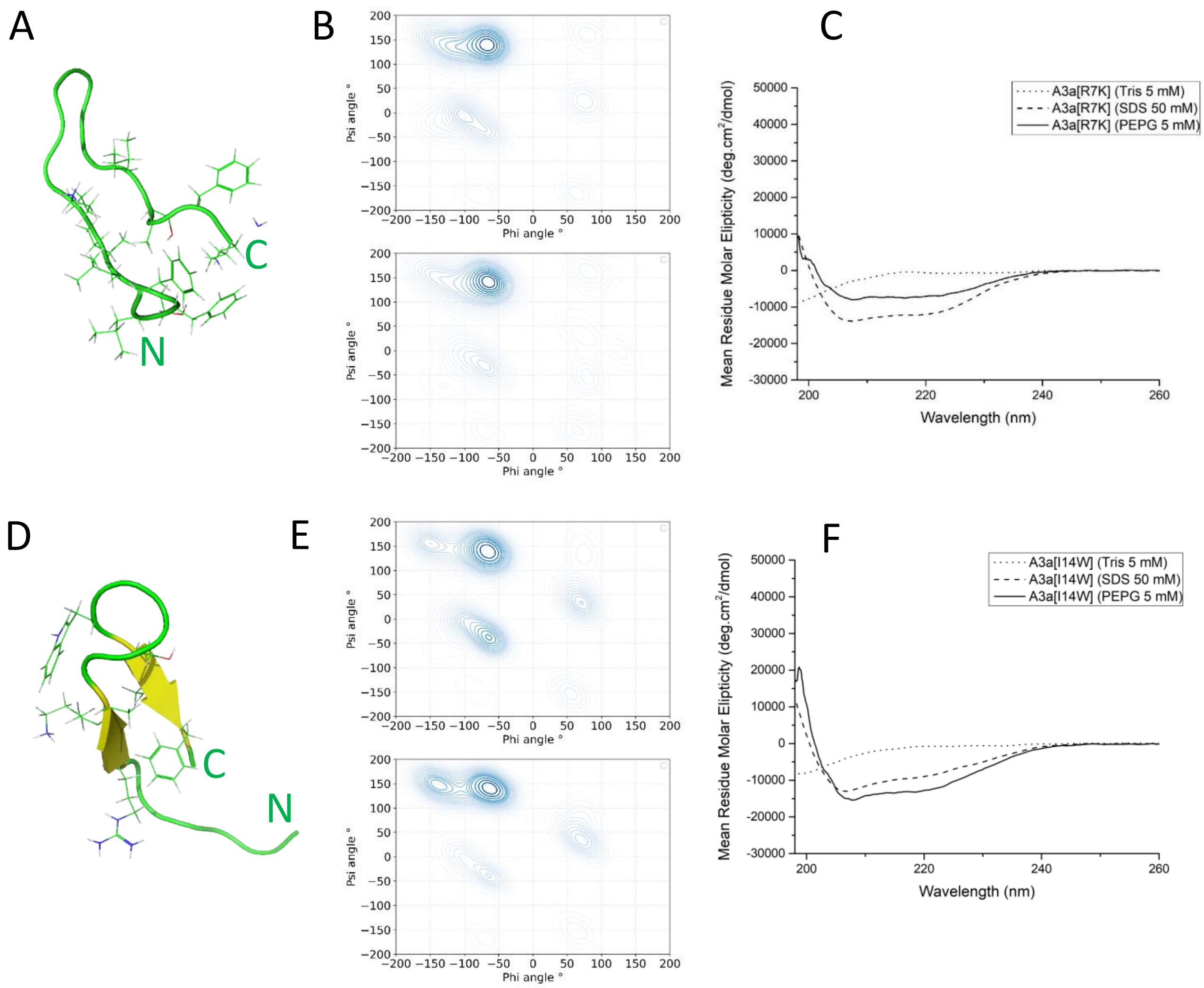


**Supplementary Figure 1. Effect of mutation on the conformation of A3 when binding to POPE/POPG lipid bilayers.** Representative snapshots of one peptide from each of the duplicate MD simulations (A, D), Ramachandran plots for the last 20 ns of duplicate 200 ns simulations averaged over four peptides (B, E) and far-UV CD spectra (C, F) for A3a[R7K] (A-C) and A3a[I14W] (D-F).

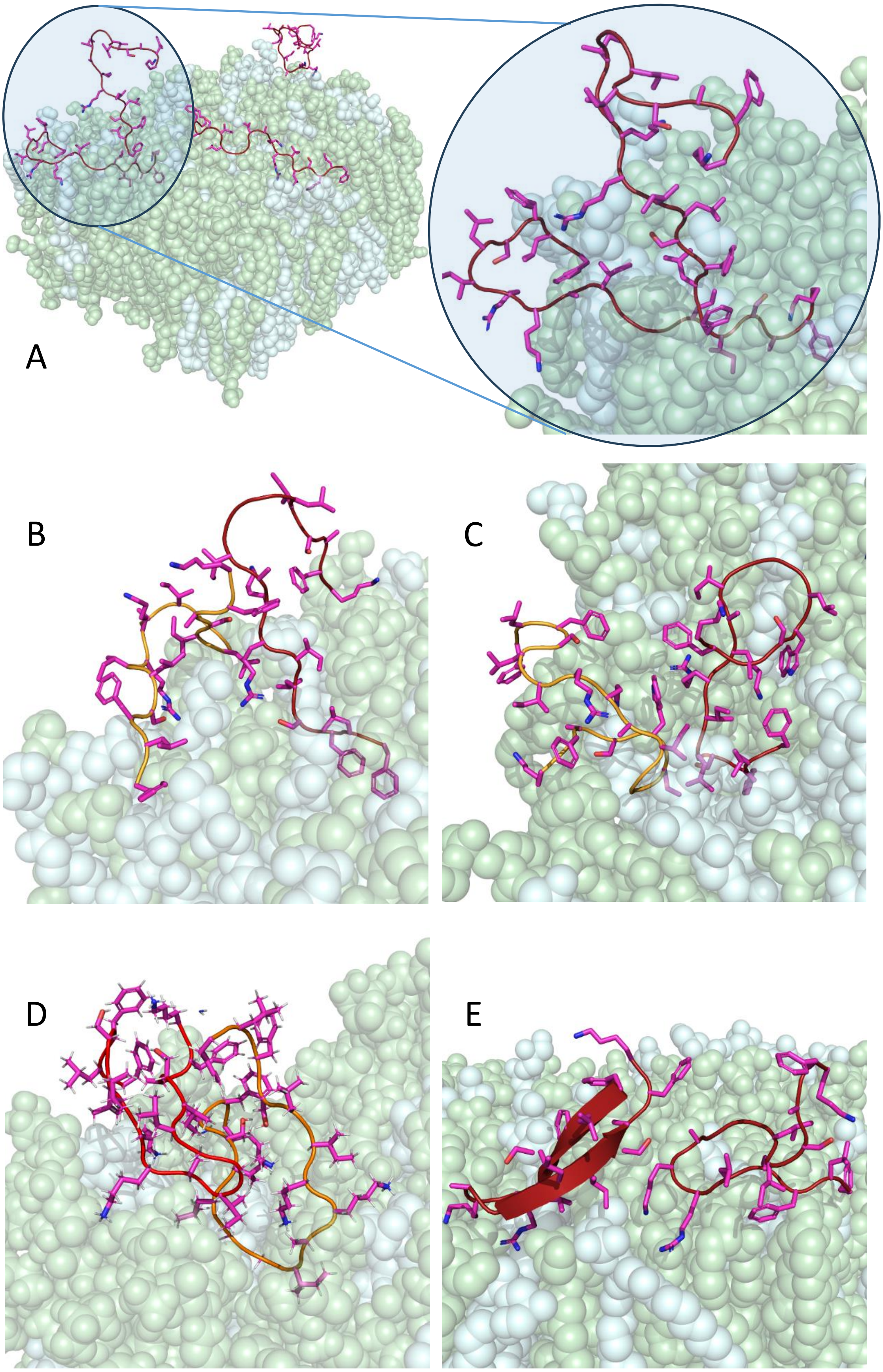


**Supplementary Figure 2. Snapshots of A3 and its analogues when binding to POPE/POPG lipid bilayers**. Representative snapshots of each of the duplicate MD simulations, at 200 ns, for A3 (A), A3a (B), A3a[I14W] (C), A3a[R7K] (D) and A3a[I(6,10)del] (E). POPE lipids are shown in green.

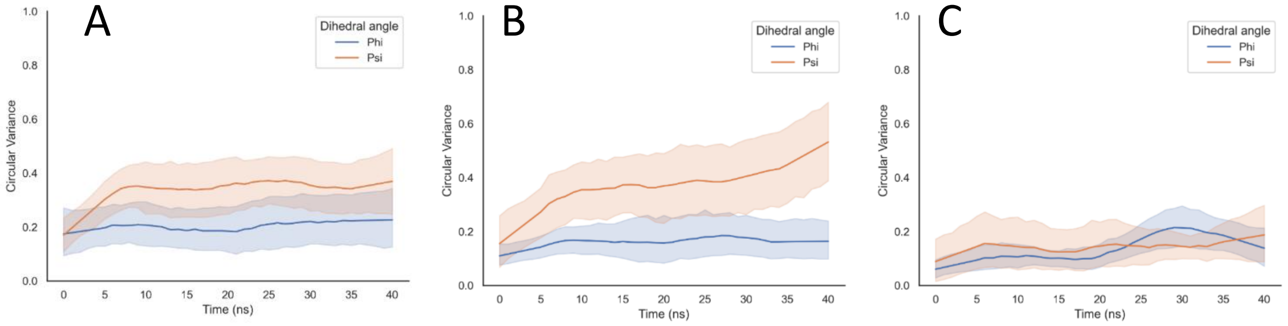


**Supplementary Figure 3. Deletion of Ile6 and Ile10 attenuates conformational flexibility when binding to POPE/POPG lipid bilayers (replication).** Circular variance for phi and psi Ramachandran angles averaged over four peptides over the 200 ns duration (A-C) for repeated MD simulations for A3 (A), A3a (B) and A3a[I(6,10)-del](C).

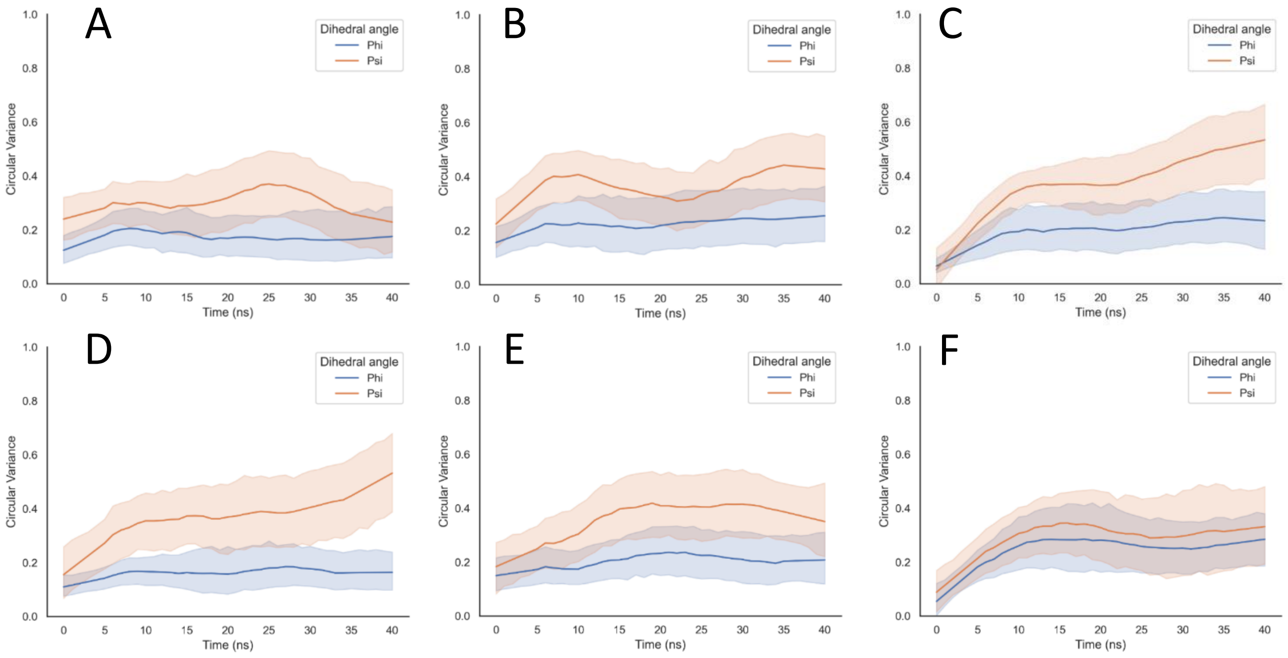


**Supplementary Figure 4. Mutation of A3a does not affect conformational flexibility when binding to POPE/POPG lipid bilayers.** Circular variance for phi and psi Ramachandran angles averaged over four peptides over the 200 ns duration for duplicate MD simulations for A3a (A, D), A3a[R7K] (B, E) and A3a[I14W] (C, F).

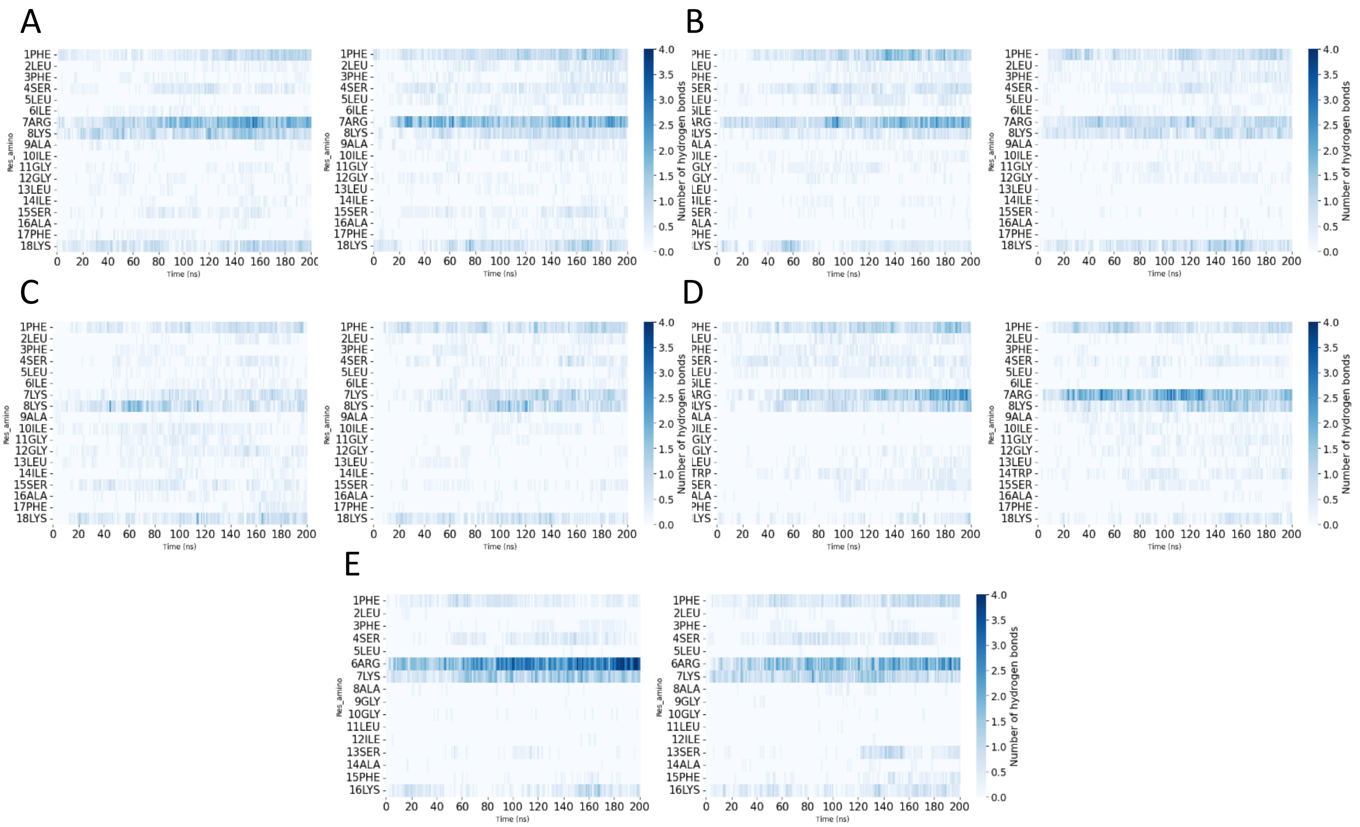


**Supplementary Figure 5. Lipid interaction when binding to POPE/POPG lipid bilayers.** Duplicate time-resolved peptide-lipid hydrogen bonding heatmaps for A3 (A), A3a (B), A3a[R7K] (C), A3a[I14W] (D) and A3a[I(6,10)-del] (E).

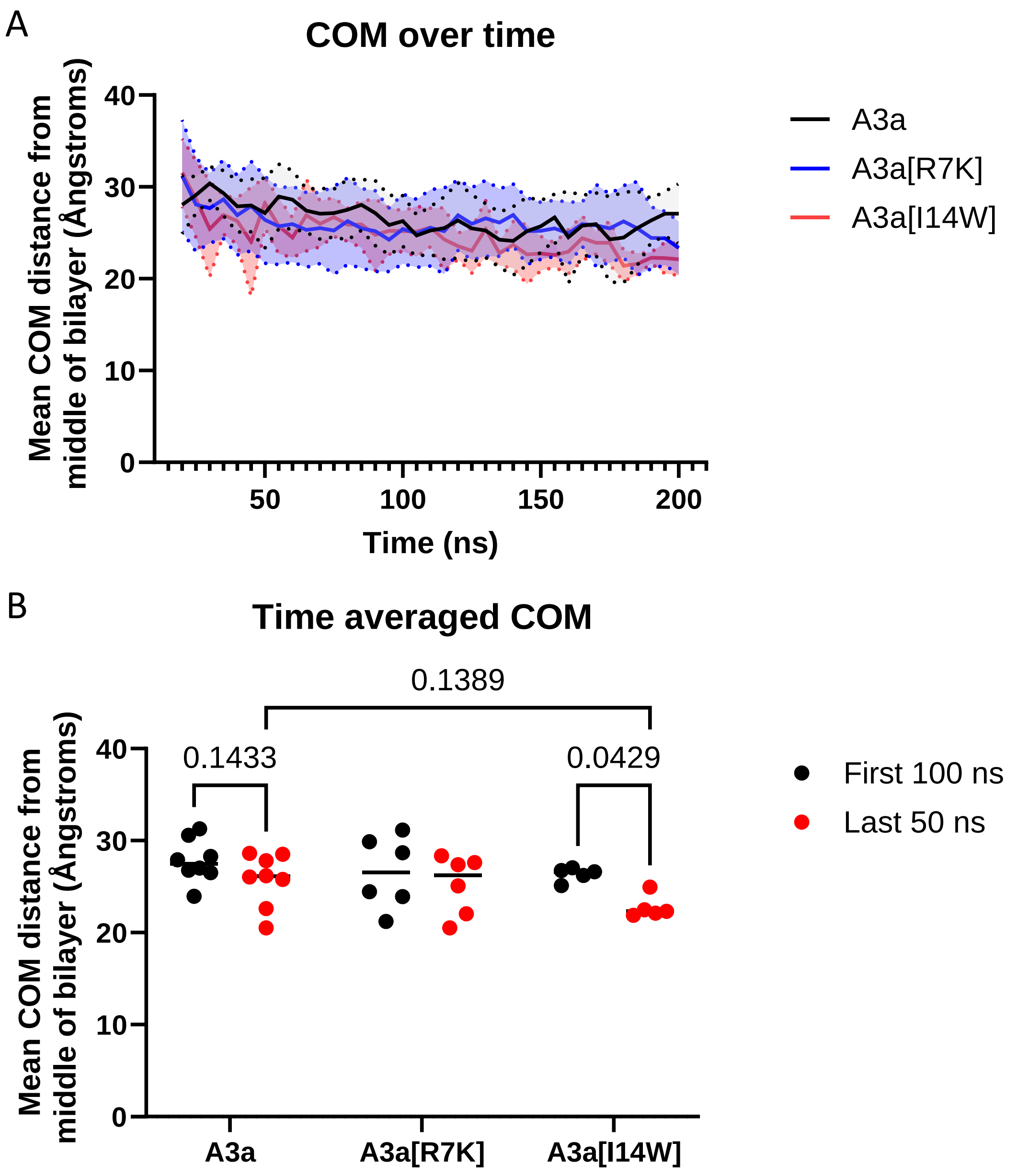


**Supplementary Figure 6. Centre of Mass (COM) analysis for peptide binding to POPE/POPG lipid bilayers.** COM distances from the middle of the bilayer are shown as an average of all peptides in two replicate simulations over time (A) or per peptide, averaged over the first 100 or last 50 ns of replicate 200 ns simulations for A3a, A3a[R7K] and A3a[I14W] (B). Peptides that cross the periodic boundary – one peptide in each replicate for A3a[R7K] and three divided between the two replicates for A3a[14W]- are excluded from this analysis. Statistical analysis is by two-way ANOVA with Tukey correction for multiple comparisons. Only comparisons with $p < 0.2$ are shown.

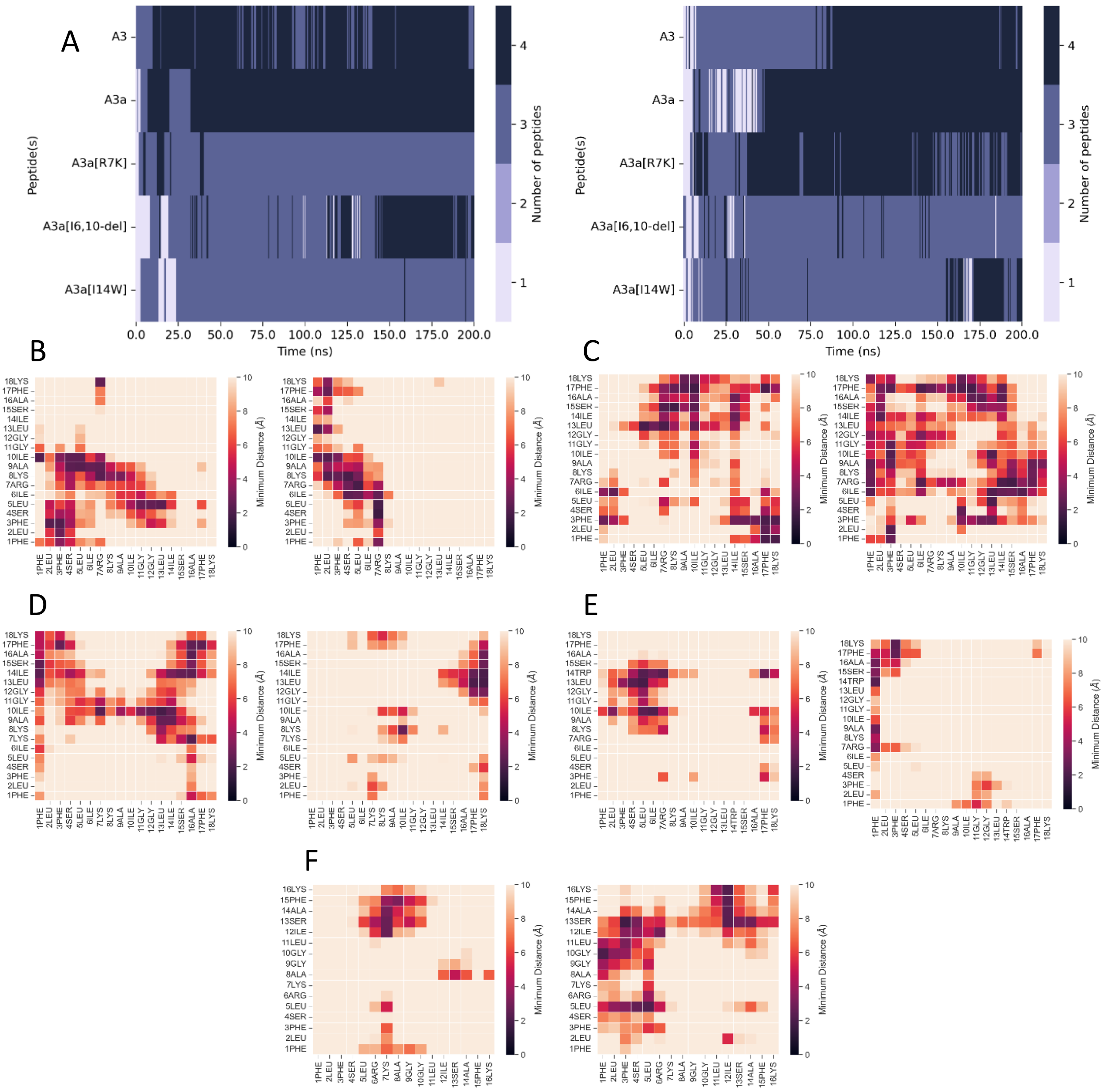


**Supplementary Figure 7. Peptide aggregation and lipid interaction when binding to POPE/POPG lipid bilayers.** Residency of A3, A3a and its three analogues as tetramers, trimers, dimers or monomers for four peptides binding to 512 POPE:POPG (3:1) lipid bilayers over the 200 ns duration of duplicate simulations (A). Time averaged aggregation matrices (B-F) for A3 (B), A3a (C), A3a[R7K] (D). A3a[I14W] (E) and A3a[I(6,10)-del] (F). Aggregation data is compromised by consistent crossing of the periodic boundary conditions by A3a[R7K] and A3a[I14W] with either one or two of four peptides unavailable for aggregation.

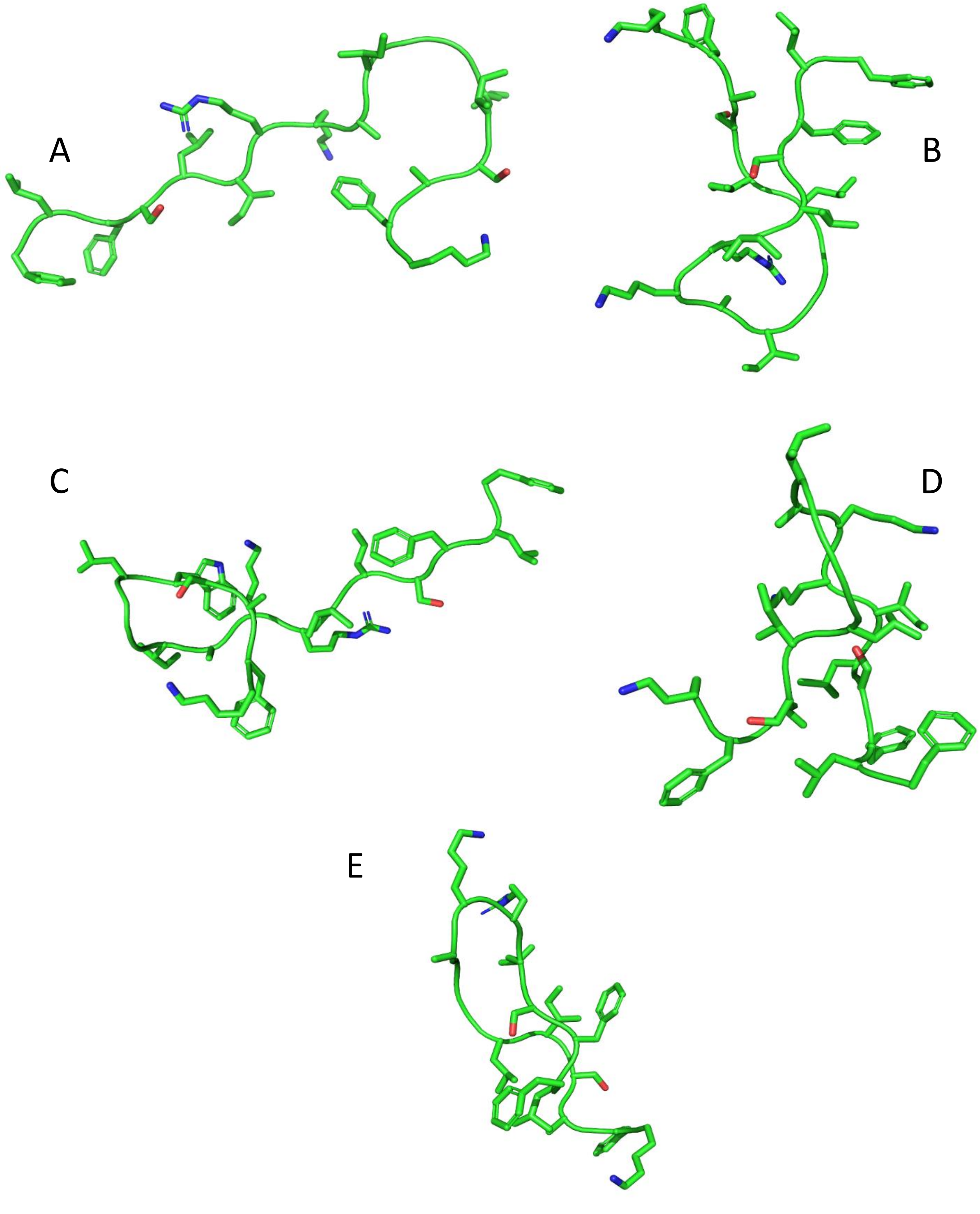


**Supplementary Figure 8. Starting structures for A3 and its analogues for the MD simulations.** A3 (A), A3a (B), A3a[I14W] (C), A3a[R7K] (D) and A3a[I(6,10)del] (E).
